# A Discriminative Enhancement and Selective Fusion Method for Low-Light Cross-Spectral Object Detection

**DOI:** 10.3390/s26092684

**Published:** 2026-04-26

**Authors:** Ping Yang, Jiahui Jiang, Yujie Zhang

**Affiliations:** School of Electronic Information and Artificial Intelligence, Shaanxi University of Science and Technology, Xi’an 710021, China; yangping@sust.edu.cn (P.Y.); zhangyujie@sust.edu.cn (Y.Z.)

**Keywords:** low-light object detection, cross-spectral fusion, retinex enhancement, selective fusion

## Abstract

Under low-light conditions, visible-spectrum images are prone to detail loss and contrast degradation, which substantially limits object detection performance. Although infrared imagery can provide complementary cues, direct fusion often introduces noise interference and thus undermines detection stability. To address this issue, this paper proposes a discriminative enhancement and selective fusion method for low-light cross-spectral object detection. Specifically, a task-oriented discriminative Retinex enhancement module is introduced at the front end to mitigate illumination interference while strengthening structural information. Meanwhile, a spectral-selective cross-scale fusion module is designed to suppress noise propagation through adaptive weighting and cross-scale interaction. In addition, mutual information loss and cross-scale consistency constraints are incorporated to enhance cross-spectral feature representation and prediction stability. Experimental results on multiple public datasets demonstrate that the proposed method can consistently improve the accuracy and robustness of object detection under low-light conditions.

## 1. Introduction

In recent years, with the development of deep convolutional neural networks and the emergence of single-stage object detectors such as YOLO, object detection technology has achieved significant progress in both accuracy and efficiency, and has been widely applied in scenarios such as intelligent transportation and video surveillance. However, under low-light conditions, visible-spectrum images are often affected by contrast degradation, noise amplification, and structural detail deterioration, which lead to unstable feature extraction and consequently significantly weaken detection performance [1,2,3]. How to obtain stable and discriminative feature representations under low-light conditions remains a key problem that urgently needs to be addressed in the field of object detection.

To alleviate the limitations of visible-spectrum imaging under low-light conditions, researchers have gradually introduced cross-spectral imaging modalities such as thermal infrared and near-infrared. Thermal infrared imaging is insensitive to illumination variations, whereas near-infrared imaging can preserve relatively clear structural information under weak illumination. Existing studies have shown that the joint modeling of cross-spectral information helps improve the robustness of object detection in nighttime and low-light scenes [4,5,6]. Meanwhile, the introduction of cross-spectral datasets such as KAIST and LLVIP has provided an important foundation for evaluating related methods in real-world complex scenarios [7,8].

Although cross-spectral information exhibits clear complementary advantages, effectively fusing heterogeneous modal features remains challenging. Existing methods mostly adopt early-fusion or mid-fusion strategies, which generally assume that features from different spectra and scales have similar reliability. Under low-light conditions, however, this assumption often does not hold. As a result, the fusion process may amplify noise interference and further affect the stability of detection results [9,10,11]. In addition, although a large number of low-light image enhancement methods based on Retinex theory or deep learning can improve visual quality, their optimization objectives are primarily oriented toward human perception and are not necessarily beneficial to the learning of discriminative features for detection tasks [12,13].

At the model training level, even when the network architecture is reasonably designed, distribution discrepancies of cross-spectral features across different scales and modalities may still lead to inconsistent predictions. Existing studies have shown that introducing constraint mechanisms such as mutual information maximization and consistency learning helps stabilize multimodal feature distributions and improve model robustness [14,15]. However, their systematic application to low-light cross-spectral object detection remains insufficiently explored.

However, existing cross-spectral detection methods often involve a clear trade-off among detection accuracy, model complexity, and deployment friendliness. Although some dual-backbone or dual-branch methods can achieve relatively high detection accuracy, they are usually accompanied by a larger parameter scale and higher inference cost, making them less suitable for practical deployment in resource-constrained scenarios. In contrast, more compact mid-level fusion frameworks have inherent advantages in maintaining lower complexity and higher inference efficiency, yet their fusion stability and feature representation capability still have room for further improvement. Based on the above analysis, this paper proposes a discriminative enhancement and selective fusion method for low-light cross-spectral object detection, which systematically models the detection stability problem from three aspects: input enhancement, feature fusion, and training constraints. Experimental results on multiple low-light cross-spectral datasets, including KAIST, FLIR-Aligned, and LLVIP, demonstrate that the proposed method consistently outperforms the baseline model under different cross-spectral input configurations. The main contributions of this paper are summarized as follows:

⮚ A task-driven framework for low-light cross-spectral object detection is proposed, which achieves collaborative optimization of feature degradation, fusion instability, and training inconsistency.
⮚ A discriminative Retinex enhancement module (TARD) is designed to improve the discriminability of detection-oriented features under low-light conditions.
⮚ A spectral-selective cross-scale fusion module (SSCF) is proposed to enhance the robustness of cross-spectral fusion through adaptive spectral and scale weighting.
⮚ Mutual information loss and consistency constraints are introduced during training to further stabilize the learning process of cross-spectral features.
⮚ The comprehensive advantages of the proposed method in terms of accuracy, stability, and efficiency are validated through extensive experimental evaluations on multiple datasets.

## 2. Related Work

### 2.1. Research on Low-Light Object Detection

Existing low-light object detection methods can generally be divided into two categories: image-enhancement-based detection methods and detection-model-optimization-based methods. Image-enhancement-based methods usually improve the quality of detection inputs by enhancing the brightness and contrast of low-light images. Representative works include the illumination-reflectance decomposition model proposed by Fu et al. [16] based on Retinex theory, as well as the deep Retinex decomposition network (RetinexNet) proposed by Wei et al. [17], which alleviates low-light degradation by explicitly or implicitly modeling the illumination component [18]. Although such methods have achieved certain improvements in visual quality, their optimization objectives are primarily oriented toward human visual perception. Therefore, the enhanced results are not necessarily consistent with the discriminative features required for object detection tasks and may even introduce additional noise interference in some scenarios, thereby negatively affecting detection performance [3].

Another line of research mainly focuses on improving the detection model itself to enhance its adaptability to low-light scenes. For example, Jiang et al. [19] incorporated a low-light enhancement branch into the YOLO framework and improved object detection performance in nighttime scenes through end-to-end training. Li et al. [20], from the perspective of frequency-domain modeling, improved detection performance in low-light environments by suppressing low-light noise and strengthening structural information. Such methods alleviate performance degradation under low-light conditions to a certain extent, but they still mainly rely on visible-spectrum images as the primary input source. In scenarios with extremely low illumination or severely degraded lighting conditions, relying solely on visible-spectrum imaging is often insufficient to compensate for the loss of critical information. As a result, further improvement in detection performance remains significantly constrained [21].

### 2.2. Research on Cross-Spectral and Multimodal Object Detection

To address the insufficient information representation of visible-spectrum images under low-light conditions, cross-spectral object detection methods introduce imaging modalities such as thermal infrared and near-infrared to provide complementary information for target perception in complex illumination environments, thereby enhancing the environmental adaptability of detection models. According to the stage at which multimodal features are involved in fusion, existing methods can generally be categorized into three types: early fusion, mid-level fusion, and late fusion [22,23,24].

Early-fusion methods typically concatenate data from different spectral bands at the input layer or along the channel dimension, enabling the network to directly perform joint feature learning. Such methods are relatively simple in structure and usually incur low computational cost [25]. However, they generally assume that different spectra have approximately similar information reliability across various scenes, while paying limited attention to the differences in imaging mechanisms and noise distributions among different modalities. Therefore, under low-light conditions, early fusion tends to introduce noise from low-quality modalities into the network together with useful information, thereby interfering with the subsequent detection process.

Mid-level fusion methods usually introduce attention mechanisms, cross-modal interaction modules, and similar structures during the feature extraction stage or feature pyramid stage to enhance the representation capability of multimodal features [24,26,27]. Compared with early fusion, such methods usually achieve better detection performance. However, existing studies show that many of these methods still adopt weighting or interaction strategies that are independent of modality quality. When a certain spectral band undergoes severe degradation under low-light conditions, its noisy information may still be propagated to subsequent layers along with multi-scale features, thereby affecting the stability of detection results.

In recent years, YOLO-based single-stage cross-spectral detection frameworks have gradually attracted increasing attention. Wan et al. [28] proposed YOLOv11-RGBT, which realizes multimodal object detection within a unified framework and achieves a certain balance between detection accuracy and inference efficiency. However, such methods generally lack explicit modeling of the reliability of different spectra during the fusion process. When some modalities suffer from severe degradation in low-light environments, simple fusion strategies are often insufficient to effectively suppress noise propagation, leading to fluctuations in detection performance under complex scenes [28,29].

In addition to CNN-based fusion frameworks, recent research has increasingly explored transformer-based and unified RGB-IR detection paradigms. For example, transformer-based multimodal detectors improve cross-modal interaction through stronger global modeling and complementary feature calibration, thereby enhancing representation capability in complex scenes. Meanwhile, more recent unified frameworks such as UniRGB-IR [30] introduce adapter tuning into RGB-IR downstream tasks by leveraging RGB-pretrained foundation models and lightweight modality adapters, showing that parameter-efficient transfer has become a promising direction for multimodal perception. However, these methods often rely on relatively strong backbones, transformer architectures, or unified large-scale modeling strategies, which may improve representation power at the cost of higher structural complexity or reduced deployment friendliness. In contrast, for low-light cross-spectral object detection, how to jointly maintain feature discriminability, fusion stability, and practical efficiency in a compact framework remains insufficiently explored.

### 2.3. Multimodal Feature Fusion and Training-Level Constraint Methods

In addition to fusion strategies at the network-architecture level, the role of training-stage constraints in multimodal feature learning has also attracted increasing attention. In terms of structural design, attention mechanisms and cross-modal feature interaction modules have been widely used to improve the information fusion capability among different modalities [26,27]. However, relying solely on network structures for feature modeling may still lead to problems such as modality bias and inconsistent scale representations in complex low-light scenes [28].

At the same time, recent multimodal frameworks have also benefited from stronger model designs and more advanced fusion strategies [31]. Although such designs can improve generalization and representation capacity, they do not directly resolve the instability caused by low-light degradation, modality reliability differences, and inconsistent cross-scale responses in compact detection frameworks. Therefore, for low-light cross-spectral object detection, explicit task-oriented constraints on enhanced features and fused multi-scale representations remain necessary.

From the perspective of training strategies, methods such as mutual information maximization [32], contrastive learning, and consistency constraints [33,34] have been shown to help stabilize feature distributions and improve the generalization ability of models. Nevertheless, the systematic application of such training-level constraints to low-light cross-spectral object detection remains relatively insufficient. Most existing methods still primarily rely on conventional detection losses as the main optimization objective, making it difficult to simultaneously account for consistency constraints across both cross-spectral features and cross-scale features. This, to some extent, limits the detection stability of models in complex low-light scenes [35].

In summary, existing studies have made notable progress in low-light object detection, cross-spectral fusion, and multimodal feature learning. Recent methods have further explored transformer-based interaction, unified RGB-IR frameworks, and parameter-efficient transfer based on pretrained models. However, these approaches often emphasize stronger backbones or more complex unified modeling, while the joint problem of low-light degradation suppression, reliability-aware fusion, and lightweight yet stable optimization remains insufficiently addressed. Therefore, this paper develops a task-driven framework for low-light cross-spectral object detection, which improves detection robustness from three complementary aspects: input enhancement, selective fusion, and training constraints.

## 3. Method

**Figure 1:**
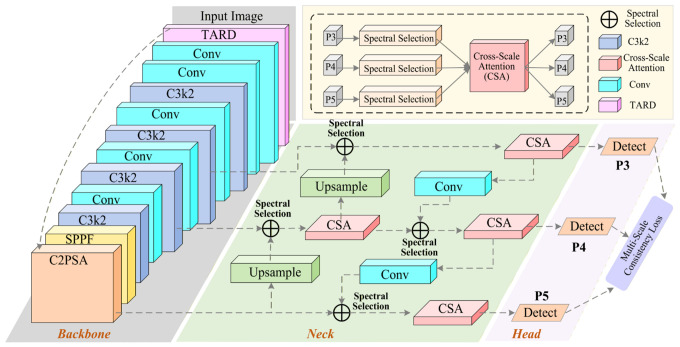
Overall architecture diagram of the proposed framework. Different colors are used to distinguish different functional modules/stages.

This section presents the proposed framework for low-light cross-spectral object detection. Built upon the YOLOv11 detector, the framework consists of three coordinated components: a task-oriented discriminative Retinex enhancement module (TARD) at the input stage, a spectral-selective cross-scale fusion module (SSCF) in the neck, and auxiliary training constraints for feature regularization. The overall architecture is illustrated in Figure 1.

In the proposed framework, a pair of cross-spectral images (e.g., RGB–thermal infrared or RGB–near-infrared) is first processed by TARD to enhance detection-relevant structural information under low-light conditions. The enhanced features are then fed into the backbone for multi-level feature extraction. After that, SSCF performs adaptive fusion across modalities and scales to generate more stable multi-scale representations for the detection heads. During training, mutual information loss and cross-scale consistency constraints are further introduced to regularize feature learning and prediction behavior. These auxiliary constraints are used only in training and do not introduce additional overhead during inference.

### 3.1. Discriminative Retinex Enhancement Module (TARD)

Under low-light conditions, visible images often suffer from degraded contrast and weakened structural cues, which are unfavorable for stable object detection. Following the Retinex-inspired view, a low-light image can be modeled as the pixel-wise product of the reflectance component and the illumination component [36]:

(1) I(x)=R(x)⊙L(x)

where ⊙ denotes element-wise multiplication.

From a practical perspective, the illumination component usually varies more smoothly in local regions, whereas reflectance-related responses tend to preserve richer structural cues such as edges, contours, and textures. This difference suggests that decomposition-inspired enhancement may help suppress illumination-dominant interference while retaining target-related details that are more useful for object detection. In addition, when the observation noise is not overwhelmingly strong, such a separation mechanism can still provide a useful basis for improving the discriminability of detection inputs under low-light conditions.

Based on this intuition, this paper introduces a task-oriented discriminative Retinex enhancement module (TARD) at the front end of the detection network. Instead of performing a handcrafted decomposition, TARD implements the enhancement process in a learnable and detection-driven manner, enabling the model to adaptively emphasize structure-relevant information while suppressing illumination-related interference. From a task-oriented viewpoint, reflectance-dominant responses are more closely associated with object boundaries, local textures, and target contours, whereas illumination mainly describes global lighting conditions. Therefore, enhancing reflectance-related information and reducing illumination disturbance is beneficial for providing more discriminative inputs to the subsequent detection network.

**Figure 2:**
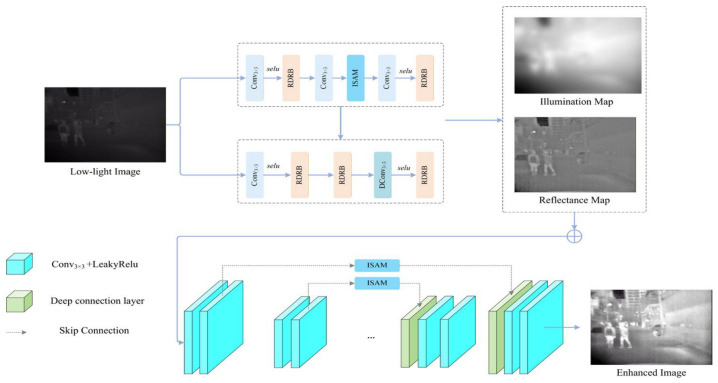
Structure of the TARD module. Different colors are used to distinguish different functional blocks, and the ellipsis indicates omitted or repeated operations for simplicity.

As shown in Figure 2, TARD performs detection-oriented Retinex decomposition and enhancement under low-light conditions. Given a cross-spectral input image, the module first extracts local features through stacked 3 × 3 convolutions and nonlinear activations, while implicitly separating illumination-dominant responses from reflectance-dominant responses. Subsequently, residual connections are used to fuse the original input with the enhanced features, thereby suppressing illumination interference while preserving structural details.

**Figure 3:**
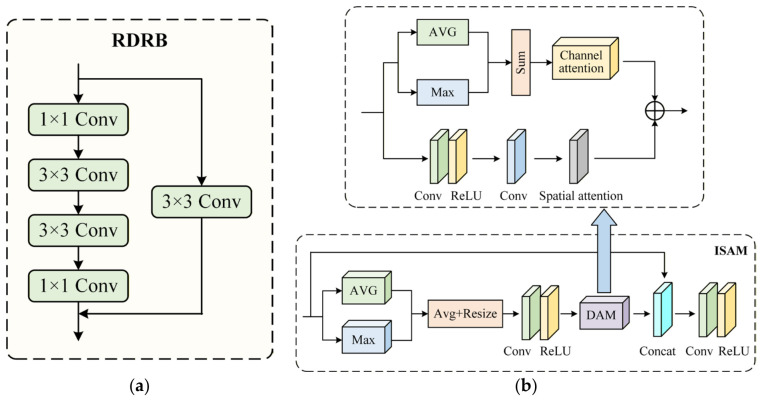
(**a**) Structure of the RDRB Module; (**b**) Structure of the ISAM Module.

Among them, the RDRB (Reflectance Detail Residual Block) adopts a residual structure composed of 1 × 1 and 3 × 3 convolutions. Specifically, the 1 × 1 convolution is used for channel-dimension adjustment, while the stacked 3 × 3 convolutions are used to capture local structural information. The residual shortcut connection helps stabilize gradient propagation and preserve detailed information of reflectance-related components during the decomposition and reconstruction stages, as shown in Figure 3a.

The ISAM (Illumination-Suppressed Attention Module) performs feature recalibration through a channel-spatial attention mechanism. Specifically, global average pooling and max pooling are first employed to extract global contextual statistical information, and effective responses are then enhanced through channel attention. Subsequently, the spatial attention branch is used to further refine feature localization. The final fused features can suppress illumination-dominant components while enhancing structure-related responses, as shown in Figure 3b.

**Figure 4:**
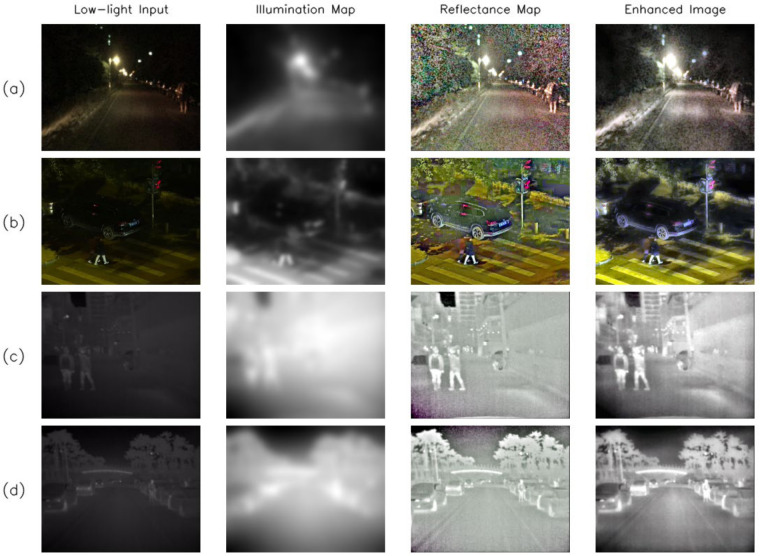
Visualization results of the illumination maps, reflectance maps, and enhanced images produced by TARD on representative low-light examples. From left to right, each row shows the low-light input, the illumination map, the reflectance map, and the enhanced image. (**a**) A nighttime road scene; (**b**) a low-light street scene with vehicles and pedestrians; (**c**) a pedestrian-dominant low-light scene; (**d**) a nighttime street scene with surrounding buildings and vehicles. The colors in the reflectance maps are used only for visualization of structural responses and do not indicate semantic categories.

To further illustrate the decomposition and enhancement behavior of TARD, multiple visual examples are presented in Figure 4. For each low-light input image, the corresponding illumination map, reflectance map, and enhanced image are shown. The results indicate that TARD can produce relatively smooth illumination estimates while preserving structure-related information in the reflectance component. Meanwhile, the enhanced outputs improve the visibility of target regions and scene structures under different low-light conditions, thereby providing more discriminative inputs for the subsequent detection network.

Finally, the enhanced features produced by TARD are fed into the backbone network for multi-level feature extraction and are jointly optimized with the subsequent spectral-selective cross-scale fusion module (SSCF). Since TARD is trained end-to-end together with the detection network, its enhancement process can adaptively serve the object detection task rather than merely satisfying visual perception requirements.

### 3.2. Spectral-Selective Cross-Scale Fusion Module (SSCF)

In low-light cross-spectral detection, features from different modalities and scales are not equally reliable. Direct multi-scale aggregation may therefore propagate noisy or redundant responses. To address this issue, we introduce a spectral-selective cross-scale fusion module (SSCF), which performs channel-wise spectral recalibration and adaptive multi-scale fusion for more stable feature aggregation.

**Figure 5:**
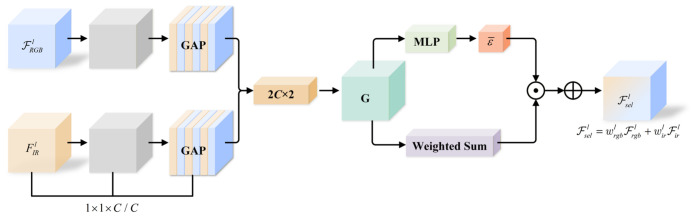
Structure of the spectral selection module. The symbol with a central dot denotes feature weighting, and the symbol with a plus sign denotes feature fusion.

The spectral selection branch first aggregates contextual information from the features through global average pooling, and then employs a lightweight multilayer perceptron (MLP) to generate reliability weights along the channel dimension, which are subsequently normalized by a Sigmoid function. The obtained weights are used to reweight features from different modalities, thereby suppressing the responses of unreliable spectral bands while enhancing the spectral information with stronger discriminative capability, as shown in Figure 5.

Let the cross-spectral feature representation output by the backbone network at the m-th scale be defined as:

(2) $$F_{m}\in {\mathbb{R}}^{C\times H_{m}\times W_{m}},m=1,\ldots ,M$$

where C denotes the number of channels and contains feature responses from different spectral modalities.

At each scale m, SSCF introduces a spectral selection mechanism along the channel dimension to characterize the relative reliability of features from different spectral modalities. First, global average pooling is applied to the feature map to aggregate contextual information:

(3) $$z_{m}=\mathrm{GAP}(F_{m})$$

Subsequently, a channel weight vector is generated through two layers of lightweight transformations:

(4) $$w_{m}=\sigma \left(W_{2}\delta \left(W_{1}z_{m}\right)\right)$$

where δ(⋅) and σ(⋅) denote the ReLU and Sigmoid functions, respectively. The feature representation after spectral-selective weighting can be expressed as:

(5) $${\tilde{F}}_{m}=w_{m}⊙F_{m}$$

This process can suppress the responses of noisy spectral bands and emphasize spectral information with stronger discriminative capability, thereby avoiding the problem of treating features from different modalities equally, as is commonly done in traditional fusion methods.

**Figure 6:**
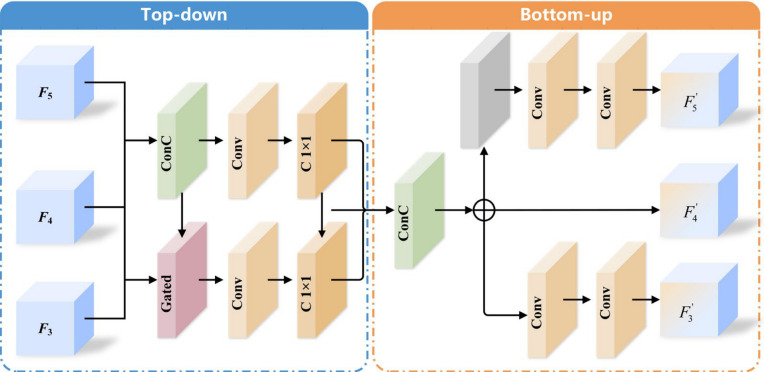
Structure of the Cross-Scale Fusion Module. The symbol with a plus sign denotes feature fusion of different branches.

After spectral selection is completed, SSCF further introduces a cross-scale attention mechanism to model the dependency relationships among features at different scales. As shown in Figure 6, the cross-scale fusion stage adopts a bidirectional structure consisting of top-down and bottom-up pathways. Features at different resolution levels are first unified in scale through interpolation-based alignment and concatenation operations, and are then refined by convolution. Unlike traditional FPN or BiFPN structures, which use spatially invariant scalar weights for fusion, SSCF introduces spatially adaptive attention weights to dynamically regulate the contribution of features at different scales at different spatial locations, thereby achieving more stable and discriminative multi-scale fusion.

First, features at all scales are uniformly aligned to the highest-resolution level:

(6) $${\hat{F}}_{m}=U_{m}\left({\tilde{F}}_{m}\right)$$

where $U_{m}(\cdot )$ denotes the bilinear interpolation operation. Subsequently, a cross-scale attention subnetwork is employed to generate spatially varying scale weights:

(7) $${\left\{G_{m}\right\}}_{m=1}^{M}={\mathrm{Softmax}}_{m}\left(\phi ({\hat{F}}_{1},\ldots ,{\hat{F}}_{M})\right)$$

where $G_{m}\in {\mathbb{R}}^{1\times H\times W}$ denotes the contribution of the m-th scale to the fusion result at spatial position.

The final fused feature is computed as:

(8) $$F_{\mathrm{fused}}=\sum_{m=1}^{M}G_{m}⊙{\hat{F}}_{m}$$

The collaborative effect of spectral selection and cross-scale attention enables the network to dynamically adjust the importance of features at different scales according to target size and contextual information, thereby effectively alleviating noise propagation and scale mismatch during cross-spectral multi-scale fusion.

From the perspective of function representation capability, SSCF possesses stronger modeling capacity than traditional feature pyramid fusion methods. In structures such as BiFPN, multi-scale features are usually fused through spatially invariant scalar weights, which can be expressed as:

(9) $$F_{\mathrm{BiFPN}}(x,c)=\sum_{m=1}^{M}w_{m,c}F_{m}(x,c)$$

where the weight $w_{m,c}$ remains identical across all spatial positions x, and is therefore unable to capture the differentiated demands for scale information in different spatial regions. In contrast, SSCF adopts a spatially adaptive gating weight $G_{m}(x)$, and its fusion form can be expressed as:

(10) $$F_{SSCF}(x,c)=\sum_{m=1}^{M}G_{m}(x){\hat{F}}_{m}(x,c)$$

Since SSCF introduces spatially varying weight mappings into the parameter space, this spatially adaptive weighting scheme provides SSCF with greater flexibility than spatially invariant fusion weights, which will help better model spatially varying scale preferences in low-light cross-spectral scenes. Because the reliability of spectral information often varies significantly across different spatial regions and different scales, this property is particularly important in low-light cross-spectral scenarios.

**Figure 7:**
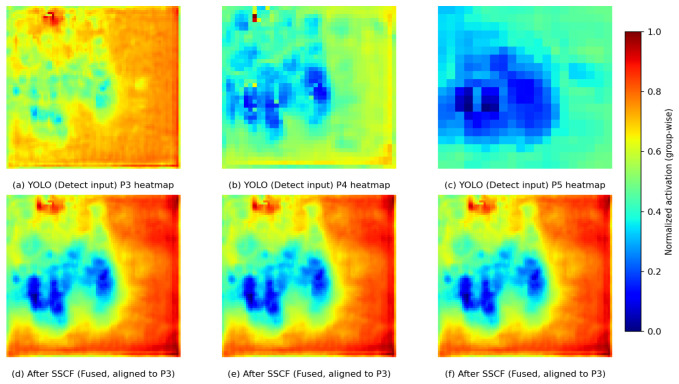
Visualization Results of Multi-Scale Feature Responses Before and After SSCF.

As shown in Figure 7, the first row presents the heatmaps of the three-scale detection-head input features (P3, P4, and P5) output by the original YOLO neck, while the second row shows the feature responses after SSCF fusion and alignment to the P3 resolution. To avoid visual bias caused by differences in feature magnitude ranges, group-wise min-max normalization is adopted in this paper: the original multi-scale features (P3–P5) are jointly normalized, whereas the fused features are normalized separately. It can be observed that SSCF suppresses background noise activation while enhancing cross-scale consistent responses in target regions, thereby providing more stable and more discriminative feature representations for cross-spectral object detection.

In practical implementation, SSCF is embedded into the neck of the base detector and is jointly trained end-to-end together with TARD and the detection heads. While preserving the efficient inference characteristics of a single-stage detector, SSCF provides the detection heads with more discriminative and stable multi-scale feature representations, thereby establishing a reliable feature foundation for low-light cross-spectral object detection.

### 3.3. Loss Function Design

In addition to the architectural improvements introduced above, the proposed framework further incorporates auxiliary training constraints for optimization. Specifically, on top of the standard detection loss, a mutual information constraint and a cross-scale consistency constraint are introduced. The mutual information term is designed to strengthen the semantic alignment between the enhanced features generated by TARD and the fused features output by SSCF, while the cross-scale consistency term is used to regularize prediction agreement among different detection scales during training. By jointly considering these terms, the overall objective promotes more discriminative feature learning and more stable prediction behavior in low-light cross-spectral object detection.

The basic detection objective follows the conventional loss function in the YOLO framework, which consists of a bounding-box regression term, an objectness confidence term, and a classification term, and can be formulated as:

(11) $$L_{det}=\lambda_{box}L_{box}+\lambda_{obj}L_{obj}+\lambda_{cls}L_{cls}$$

where λ denotes the weighting coefficient of each component.

To strengthen the semantic consistency between the enhanced features generated by the TARD module and the fused features output by SSCF, this paper maximizes the mutual information between them. Let R denote the output features of TARD and $F_{fused}$ denote the fused features of SSCF. Their mutual information is defined as:

(12) $$I(R;F_{fused})=E\left[\log\frac{p(R,F_{fused})}{p(R)p(F_{fused})}\right]$$

Since directly estimating mutual information in a high-dimensional feature space is intractable, this paper adopts an InfoNCE-based neural estimation method. The feature maps are mapped into embedding vectors r and f through global pooling, and the following loss function is minimized:

(13) $$L_{\mathrm{MI}}=-E\left[\log\frac{\exp\left(\mathrm{sim}(r,f)/\tau \right)}{\sum_{k}\exp\left(\mathrm{sim}(r,f_{k})/\tau \right)}\right]$$

where sim(⋅) denotes the cosine similarity function, and τ is the temperature coefficient. This constraint encourages the enhanced features and fused features corresponding to the same sample to remain close to each other in the embedding space, while increasing the distance between different samples, thereby promoting the learning of discriminative representations that are spectral-invariant and relevant to the detection task.

On the other hand, although SSCF can adaptively regulate the importance of features at different scales, inconsistent predictions may still arise among detection heads at different scales in extremely low-light scenes, thereby affecting training stability and generalization ability. To address this issue, this paper introduces a cross-scale consistency constraint during training to align the prediction results of different-scale outputs. Let $P_{m}$ denote the class probability map produced by the detection head at the m-th scale. After spatial alignment, the consistency loss is defined as:

(14) $$L_{\mathrm{cons}}=\sum_{m\neq n}{\left\|U(P_{m})-U(P_{n})\right\|}_{2}^{2}$$

where U(⋅) denotes the interpolation operation used to unify the resolution. This constraint can also be applied at the feature level, namely:

(15) $$L_{\mathrm{cons}}^{\mathrm{feat}}=\sum_{m\neq n}{\left\|{\hat{F}}_{m}-{\hat{F}}_{n}\right\|}_{2}^{2}$$

Thereby directly constraining the consistency of fused features at different scales in terms of spatial structure.

By jointly considering the above loss terms, the final training objective can be written as:

(16) $$L=L_{\det}+\lambda_{\mathrm{MI}}L_{\mathrm{MI}}+\lambda_{\mathrm{cons}}L_{\mathrm{cons}}$$

where $\lambda_{\mathrm{MI}}$ and $\lambda_{\mathrm{cons}}$ are weighting coefficients, whose values will be analyzed through experiments in Section 4.

## 4. Experiments

### 4.1. Experimental Datasets

This paper utilizes three multispectral object detection datasets to systematically evaluate the effectiveness and generalization ability of the proposed method for cross-spectral object detection under low-light and complex illumination conditions. These datasets can be downloaded either from their official websites or via the links provided in the corresponding GitHub documentation. A brief introduction to each dataset is given below:

FLIR-Aligned [37]: This dataset is a visible-infrared aligned version reorganized from the original FLIR ADAS dataset. It contains aligned visible-light and thermal infrared image pairs, with annotations mainly provided for three object categories: person, car and bicycle. The FLIR-Aligned version includes 5142 aligned image pairs, among which approximately 4129 pairs are used for training and 1013 pairs are used for testing/evaluation. FLIR-Aligned is commonly used in multimodal object detection research, especially for evaluating detection performance in nighttime, low-light, and complex traffic scenarios.

KAIST [7]: The original KAIST dataset was captured using visible-spectrum and long-wave infrared (LWIR) sensors. In this study, the reannotated version provided by Li is adopted, in which only pedestrian targets are labeled. It contains a total of 8956 training pairs and 2252 validation pairs, making it well suited for cross-spectral pedestrian detection research, especially in complex scenarios such as nighttime and low-light environments.

LLVIP [8]: This dataset was acquired using visible-light and infrared cameras and contains 15,488 image pairs, with annotations provided for the pedestrian category. The image resolution is 640×512 pixels, and the image pairs are pre-registered. The dataset is divided into 12,025 training pairs and 3463 testing pairs. It is mainly used for low-light vision tasks, such as image fusion and object detection.

### 4.2. Implementation Details and Evaluation Metrics

The experiments in this paper were conducted on the Windows 11 operating system with an NVIDIA RTX 4090 GPU, and the implementation was built based on PyTorch 2.1.2. The initial learning rate was set to 0.01, the weight decay was 0.001, and the batch size was set to 10. The number of training epochs was 120 for the FLIR dataset, 120 for the KAIST dataset, and 120 for the LLVIP dataset. The input image size was set to 640×640 pixels.

The performance of the network is mainly evaluated based on the mean Average Precision (mAP) during training and the performance of the trained network on the validation set. A higher mAP value indicates stronger detection performance of the model. The specific formulas for the evaluation metrics are as follows:

(17) $$R=\frac{TP}{TP+FN}$$

(18) $$P=\frac{TP}{TP+FP}$$

(19) $$AP_{i}=\int_{0}^{1}P_{i}(R_{i})dR_{i}=\sum_{k=0}^{n}P_{i}(k)\Delta R_{i}(k)$$

(20) $$mAP=\frac{1}{C}\sum_{c=1}^{C}AP_{i}$$

In this formula, TP denotes the number of correctly predicted samples, FP denotes the number of samples incorrectly predicted as positive, and FN denotes the number of missed samples. According to different IoU threshold criteria, mAP can be divided into mAP50 and mAP50:95. Specifically, mAP50 refers to the mAP computed at an IoU threshold of 0.5, whereas mAP50:95 denotes the average mAP calculated over IoU thresholds ranging from 0.5 to 0.95 with a step size of 0.05.

### 4.3. Hyperparameter Analysis of Loss Weights

To analyze the influence of the weight settings of the mutual information constraint and the cross-scale consistency constraint on detection performance, this paper conducts hyperparameter sensitivity experiments on $\lambda_{\mathrm{MI}}$ and $\lambda_{\mathrm{cons}}$ in the loss function. The experimental results are presented in Table 1.

As can be observed from Table 1, when $\lambda_{\mathrm{MI}}$ and $\lambda_{\mathrm{cons}}$ are set to relatively small values, the influence of the additional constraints on network optimization is limited, and the improvement in detection performance is not obvious. As the weights gradually increase, the model is able to make better use of the mutual information constraint to enhance the semantic consistency between the enhanced features and the fused features, while the cross-scale consistency constraint suppresses fluctuations among predictions at different scales, thereby yielding more stable and more discriminative feature representations and correspondingly improving detection accuracy. However, when the weights continue to increase, the performance gain tends to saturate and may even exhibit a slight decline. This indicates that excessively strong regularization constraints may interfere, to some extent, with the optimization of the main detection task, thereby weakening the model’s direct learning ability for object localization and classification.

In this paper, $\lambda_{\mathrm{MI}}$ = 0.7 and $\lambda_{\mathrm{cons}}$ = 0.7 are finally selected as the default settings for subsequent experiments. This parameter combination achieves favorable detection performance while maintaining training stability, indicating that moderate mutual information constraints and cross-scale consistency constraints are both necessary and effective for low-light cross-spectral object detection.

### 4.4. Ablation Experiments

To verify the effectiveness of each key component in the proposed cross-spectral object detection framework, this paper conducts systematic ablation experiments on the KAIST dataset by progressively introducing different improved components, including the task-oriented enhancement module (TARD), the cross-scale spectral-selective fusion module (SSCF), and the training-stage loss constraint terms. The experimental results are shown in Table 2.

As can be observed from the table, after introducing the RGB + IR dual-modal input together with the TARD module, the mAP@0.5 increases to 0.695, indicating that the task-oriented input enhancement mechanism can effectively alleviate illumination degradation and provide more discriminative feature representations for subsequent fusion. After further incorporating the SSCF cross-scale spectral-selective fusion module, the model performance continues to improve to 0.718, demonstrating that dynamically selecting reliable spectral information and suppressing redundant channels in the multi-scale feature space help enhance detection stability in complex scenes. Finally, after jointly introducing the loss constraint terms into the complete framework, the model achieves the best performance on the KAIST dataset, reaching an mAP@0.5 of 0.720, which verifies the good complementarity among the proposed modules.

These results indicate that the modules proposed in this paper exhibit clear functional complementarity and jointly constitute an effective framework for low-light cross-spectral object detection.

### 4.5. Comparison with Existing Methods

#### 4.5.1. Comparison of Detection Performance

To verify the effectiveness of the proposed method, several representative methods were selected for comparison on the FLIR-Aligned and LLVIP datasets, with mAP@0.5 and mAP@0.5:0.95 adopted as the evaluation metrics. For the proposed method, the reported results are presented as mean ± standard deviation over three runs with different random seeds.

As shown in Table 3 and Table 4, the proposed method achieves competitive detection performance on both the FLIR-Aligned and LLVIP datasets. On the FLIR-Aligned dataset, it attains an mAP@0.5 of 0.771 ± 0.003 and an mAP@0.5:0.95 of 0.392 ± 0.002, outperforming CMPD, CFR, and GAFF, while remaining competitive with CFT. On the LLVIP dataset, the proposed method achieves the best mean mAP@0.5 of 0.961 ± 0.002 and attains the same mean mAP@0.5:0.95 as UniRGB-IR, while surpassing DivFusion, GAFF, CSSA, and RSDet. These results indicate that the proposed method can provide stable and discriminative feature representations for low-light cross-spectral object detection.

It is worth noting that UniRGB-IR and the proposed method follow different technical routes. UniRGB-IR is closer to a unified RGB-IR framework based on adapter tuning and pretrained multimodal representations, whereas the proposed method is designed as a compact task-driven framework for low-light cross-spectral detection. Instead of relying on a unified large-scale representation route, the proposed method explicitly addresses low-light degradation and unstable multimodal fusion through TARD, SSCF, and auxiliary training constraints. Therefore, the comparison with UniRGB-IR suggests that a lightweight and deployment-friendly task-driven design can still achieve competitive performance against recent unified RGB-IR frameworks.

Overall, the proposed method achieves competitive performance across multiple benchmark datasets while maintaining a compact architecture. These results suggest that explicit low-light enhancement and selective fusion can provide an effective balance between detection accuracy and deployment friendliness for cross-spectral object detection.

#### 4.5.2. Efficiency and Complexity Analysis

In addition to detection accuracy, model complexity and inference efficiency are also important indicators for evaluating the practical value of a method. To this end, this paper further analyzes the proposed method in terms of the number of parameters, GFLOPs, FPS, and the average inference time per image. All models were tested under the same hardware and software environment, with the input resolution uniformly set to 640×640 and the batch size set to 1. Before formal timing, a warm-up procedure was performed, after which the average runtime of multiple forward inferences was recorded and used to calculate FPS.

As shown in Table 5, the proposed method contains 3.055 M parameters and 4.539 GFLOPs, with an average inference latency of 6.250 ms per image and a corresponding inference speed of 159.995 FPS. These results indicate that the proposed method maintains relatively low model complexity while preserving favorable real-time performance. Although the introduction of TARD and SSCF increases the model complexity compared with the baseline detector, the overall increase remains limited and does not significantly affect inference efficiency.

Combined with the quantitative detection results presented above, it can be observed that the improvement of the proposed method is not achieved simply by substantially increasing model size. Instead, the proposed method enhances low-light cross-spectral feature representation through discriminative enhancement and selective fusion within a compact framework, thereby achieving a favorable balance among detection accuracy, computational cost, and deployment friendliness. Compared with some more complex fusion frameworks, the proposed method maintains competitive detection performance while preserving relatively high inference efficiency, demonstrating good deployment friendliness and practical application potential.

#### 4.5.3. Cross-Dataset Generalization Test

In addition to the within-dataset evaluation, a preliminary cross-dataset test was further conducted to assess the robustness of the proposed method under domain shift. Specifically, two cross-dataset transfer settings were considered: training on KAIST and testing on LLVIP, and training on LLVIP and testing on KAIST, both without further fine-tuning. This experiment aims to provide an initial indication of the generalization capability of the proposed framework in unseen cross-dataset scenarios.

As shown in Table 6, the overall detection accuracy decreases compared with the corresponding within-dataset setting, which is expected due to the domain discrepancy in scene characteristics, illumination patterns, and data distribution between KAIST and LLVIP. Nevertheless, the proposed method consistently outperforms the RGB + IR baseline in both transfer directions. When trained on KAIST and tested on LLVIP, the proposed method improves mAP@0.5 from 0.292 to 0.323 and mAP@0.5:0.95 from 0.141 to 0.150. When trained on LLVIP and tested on KAIST, it further improves mAP@0.5 from 0.254 to 0.282 and mAP@0.5:0.95 from 0.134 to 0.140. These results indicate that the introduced discriminative enhancement and selective fusion mechanisms provide improved robustness against cross-dataset variations. Although this evaluation is still preliminary and limited to a small number of transfer settings, it offers additional evidence that the proposed framework has the potential to generalize beyond the training domain.

#### 4.5.4. Qualitative Comparison with the Baseline

**Figure 8:**
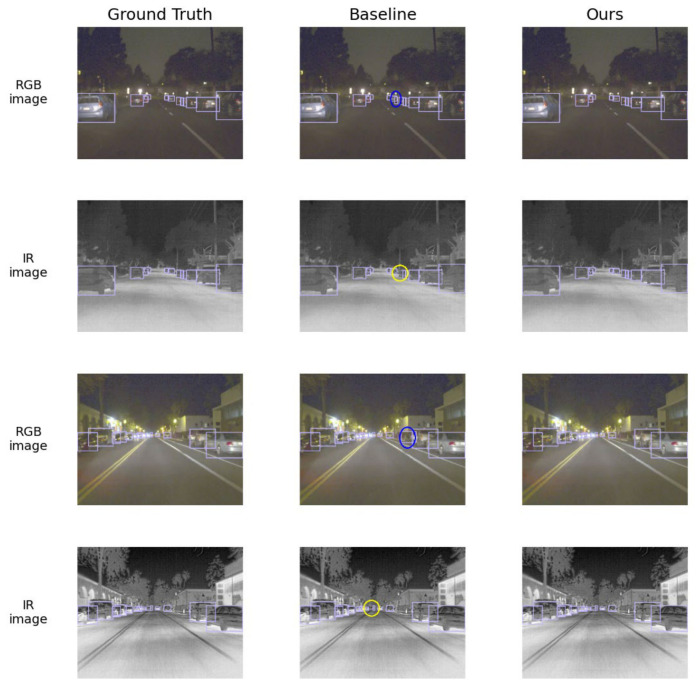
Visual comparison of detection results. The left, middle, and right columns show the ground-truth scene, the baseline results, and the results of the proposed method, respectively. Yellow ellipses indicate missed detections, while blue ellipses indicate false detections.

To intuitively demonstrate the improvement in detection performance achieved by the proposed algorithm, Figure 8 presents a comparison of the detection results between the baseline model and the proposed method under two modalities. The left column shows the ground-truth scene, the middle column shows the results of the baseline model, and the right column shows the results of the proposed method. Missed detections are marked with yellow ellipses, while false detections are marked with blue ellipses. It can be observed from the figure that, in scenes with complex illumination variations, the baseline detector still exhibits both missed detections and false detections in challenging scenes. In contrast, the proposed method integrates the advantages of the two modalities, effectively reducing missed detections and false alarms, thereby achieving superior detection performance. The visualization results provide intuitive evidence of the improvement in detection performance of the proposed method in low-light scenes.

### 4.6. Qualitative Evaluation

**Figure 9:**
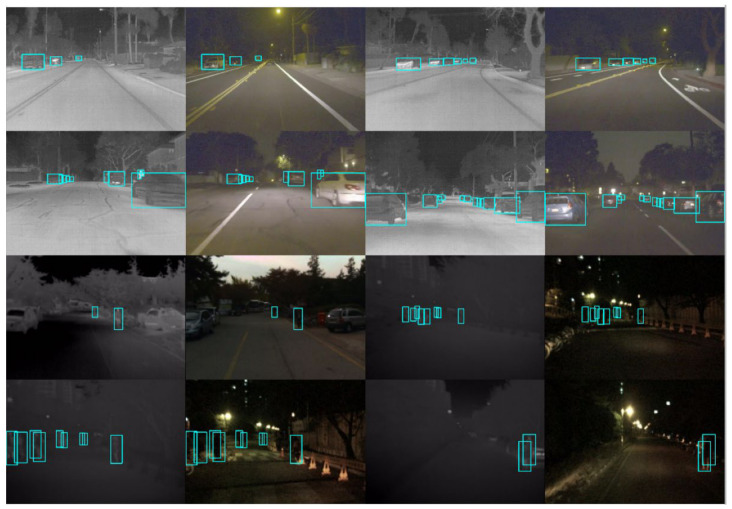
Some detection results of the proposed method on the FLIR-Aligned and KAIST datasets. The green square boxes indicate the detected object bounding boxes predicted by the proposed method.

Qualitative results of the proposed method on two multispectral datasets are presented in Figure 9. The proposed model demonstrates strong capability in object detection on multispectral images, including challenging scenarios with complex backgrounds, low object discriminability, uneven illumination, nighttime conditions, and low-angle viewpoints.

## 5. Discussion

This paper investigates low-light cross-spectral object detection and presents a task-driven framework that integrates discriminative enhancement, selective fusion, and training constraints to address feature degradation, unstable multimodal fusion, and insufficient optimization consistency under complex illumination conditions. Specifically, a task-oriented enhancement module (TARD) is introduced at the input stage, a spectral-selective cross-scale fusion module (SSCF) is designed at the feature level, and auxiliary training constraints are incorporated during optimization, enabling collaborative improvement across different stages of the network. Experiments on three public benchmark datasets, namely KAIST, LLVIP, and FLIR-Aligned, demonstrate that the proposed components contribute consistent performance gains and that the complete framework achieves competitive detection performance against representative multispectral fusion methods. In addition, repeated-run evaluation indicates that the proposed method maintains stable improvements under different random seeds, and the additional cross-dataset test further suggests its potential robustness under domain shift. Rather than pursuing the highest absolute accuracy through a substantially heavier architecture, the proposed method emphasizes a favorable balance among detection accuracy, computational cost, and deployment friendliness. Future work will further investigate broader unseen-domain generalization, more extensive comparison with recent large-scale multimodal frameworks, and improved robustness under extreme weather and complex illumination conditions, while extending the proposed framework to broader multi-sensor perception systems and intelligent transportation and smart lighting applications.

## Figures and Tables

**Table 1:** Hyperparameter Sensitivity Experiments on the KAIST Dataset.

$\lambda_{\mathrm{MI}}$	$\lambda_{\mathrm{cons}}$	mAP@0.5	mAP@0.5:0.95
0.3	0.3	0.706	0.317
0.5	0.3	0.714	0.319
0.3	0.5	0.706	0.317
0.5	0.5	0.710	0.318
0.7	0.7	0.720	0.321
0.5	0.7	0.716	0.319
0.9	0.9	0.714	0.319

**Table 2:** Ablation Experiments on the KAIST Dataset.

Method	Data	mAP@0.5	mAP@0.5:0.95
YOLOv11	RGB	0.642	0.296
YOLOv11	IR	0.673	0.301
YOLOv11	RGB + IR	0.689 ± 0.003	0.312 ± 0.001
YOLOv11 + TARD	RGB + IR	0.695	0.318
YOLOv11 + SSCF	RGB + IR	0.709	0.319
YOLOv11 + TARD + SSCF	RGB + IR	0.718	0.320
YOLOv11 + TARD + SSCF + MI	RGB + IR	0.719	0.320
YOLOv11 + TARD + SSCF + Consistency	RGB + IR	0.719	0.320
YOLOv11 + TARD + SSCF + MI + Consistency	RGB + IR	0.720 ± 0.002	0.321 ± 0.001

**Table 3:** Comparison of Results of Different Algorithms on the FLIR-Aligned Dataset.

Method	Data	mAP@0.5	mAP@0.5:0.95
CMPD [38]	RGB + IR	0.694	-
CFR [37]	RGB + IR	0.724	-
GAFF [39]	RGB + IR	0.729	0.375
CFT [40]	RGB + IR	0.779	0.396
Ours	RGB + IR	0.771 ± 0.003	0.392 ± 0.002

**Table 4:** Comparison of Results of Different Algorithms on the LLVIP Dataset.

Method	Data	mAP@0.5	mAP@0.5:0.95
DivFusion [41]	RGB + IR	0.898	0.520
GAFF [39]	RGB + IR	0.940	0.558
CSSA [42]	RGB + IR	0.943	0.413
RSDet [43]	RGB + IR	0.958	0.613
UniRGB-IR [30]	RGB + IR	0.960	0.632
Ours	RGB + IR	0.961 ± 0.002	0.632 ± 0.001

**Table 5:** Comparison of Inference Efficiency and Model Complexity.

Method	Params/M	GFLOPs	FPS	Latency/ms
YOLOv11	2.583	3.172	181.146	5.520
CFT	28.089	65.812	78.413	12.753
Ours	3.055	4.539	159.995	6.250

**Table 6:** Cross-Dataset Evaluation Results.

Train Dataset	Test Dataset	Method	mAP@0.5	mAP@0.5:0.95
KAIST	LLVIP	YOLOv11 RGB + IR	0.292	0.141
KAIST	LLVIP	Ours	0.323	0.150
LLVIP	KAIST	YOLOv11 RGB + IR	0.254	0.134
LLVIP	KAIST	Ours	0.282	0.140

## Data Availability

The datasets analyzed in this study are publicly available from their official sources, including the KAIST, FLIR-Aligned, and LLVIP datasets, as cited in the References. Processed data and implementation details are available from the corresponding author upon reasonable request.
